# Quantitative assessment of postural instability in spinocerebellar ataxia type 3 patients

**DOI:** 10.1002/acn3.51124

**Published:** 2020-07-07

**Authors:** Xia‐Hua Liu, Ying Li, Hao‐Ling Xu, Arif Sikandar, Wei‐Hong Lin, Gui‐He Li, Xiao‐Fen Li, Alimire Alimu, Sheng‐Bin Yu, Xiang‐Hui Ye, Ning Wang, Jun Ni, Wan‐Jin Chen, Shi‐Rui Gan

**Affiliations:** ^1^ Department of Rehabilitation Medicine The First Affiliated Hospital Fujian Medical University Fuzhou China; ^2^ Fujian Medical University Fuzhou China; ^3^ Department of Neurology and Institute of Neurology The First Affiliated Hospital Fujian Medical University Fuzhou China; ^4^ Fujian Key Laboratory of Molecular Neurology Fujian Medical University Fuzhou China

## Abstract

**Objective:**

Spinocerebellar ataxia type 3 (SCA3) is one of the most common hereditary neurodegenerative diseases, with balance instability as main symptom. Balance quantification is crucial for evaluating the efficacy of therapeutic interventions. However, balance evaluation in SCA3 is often subject to bias. Here, we aimed to quantitatively evaluate postural instability and investigate the relationship between postural instability and clinical characteristics in SCA3 patients.

**Methods:**

Sixty‐two SCA3 patients and 62 normal controls were recruited, and their postural balance was measured using a posturographic platform. Principal component analysis was performed as data reduction to identify postural instability factors. Multivariable linear regression was used to investigate potential risk factors for postural instability and to explore whether postural instability predicts the severity and progression of ataxia in SCA3 patients.

**Results:**

We found SCA3 patients experience postural instability characterized by significant impairment in static and dynamic stability. The condition without visual feedback was the most sensitive measure in differentiating SCA3 from controls. Regression analyses revealed that ataxia severity predicted both static (*P* = 0.014) and dynamic stability (*P* = 0.001). Likewise, along with expanded CAG repeats (*P* < 0.001), both static (*P* < 0.001) and dynamic stability (*P* < 0.001) predicted ataxia severity, but not ataxia progression.

**Interpretation:**

Our findings demonstrate the validity of using the Pro‐kin system for assessing postural instability in SCA3 patients. This type of quantitative assessment of balance dysfunction can contribute to clinical trials and balance rehabilitation in SCA3 patients.

## Introduction

Spinocerebellar ataxia type 3 (SCA3), which is also known as Machado–Joseph disease (MJD), is a common autosomal dominant neurodegenerative disorder characterized by postural imbalance, oculomotor abnormalities, dysarthria, and peripheral neuropathy.^1^, ^2^ It is regarded as the most common subtype of spinocerebellar ataxias (SCAs) in China^3^ and is caused by a pathological expansion of cytosine‐adenineguanine (CAG) trinucleotide repeats in exon10 of the *ATXN3* gene located in Chr 14q32.1, which results in the production of an abnormal expansion of polyglutamine repeats in the ataxin‐3 protein.^4^, ^5^ Normal alleles of *ATXN3* range from 12 to 44 CAG repeats, whereas the range in expanded alleles broadens to more than 50.^6^

Postural instability and gait ataxia are the most frequent initial clinical symptoms in SCA3 patients.^7^, ^8^ Postural and balance instability are commonly regarded as contributing factors to ambulatory dysfunction and risk of falls.^9^ Semiquantitative scales including the International Cooperative Ataxia Rating Scale (ICARS) and the Scale for the Assessment and Rating of Ataxia (SARA) are most commonly used to assess balance in a clinical setting.^10^, ^11^ However, research suggests that the SARA might underestimate disease severity in early phases^12^ and the ICARS may not be sensitive to short‐term changes in disease progression.^13^ Moreover, the use of several subjective components in these scales may predispose measurements to observer bias.^14^

With recent advances in techniques enabling sensitive quantification of postural characteristics, force platform posturography has been accepted as a useful technique for identifying and quantitatively evaluating balance disorders. This method can provide a visual representation of postural instability which is more objective than other methods and provided in real‐time.^15^, ^16^ The balance assessment consists of static and dynamic stability measures which provide a foundation for characterizing patients’ postural and balance instability.^17^, ^18^ Previous research suggests static stability may be correlated with disease severity.^15^ In addition, ataxia patients may experience poor body control in dynamic balance.^19^ However, the possible patterns of balance dysfunction used in posturography have not been thoroughly studied in SCA3 and the relationship between balance dysfunction and SCA3 phenotype remains unclear.

In this study, we use the Pro‐kin system to investigate and quantify posturographic indicators in order to characterize the balance dysfunction patterns associated with SCA3 patients and explore the relationships between balance dysfunction and clinical characteristics of SCA3.

## Subjects and Methods

### Study participants

Sixty‐two patients with a molecular diagnosis of SCA3 and 62 healthy participants were recruited between October 2018 and August 2019. Our inclusion criteria for SCA3 patients were (1) the presence of ataxia, with SARA scores ≥ 3, (2) definite genetic diagnosis of SCA3, (3) willingness to participate, and (4) age of 14 years or older. Exclusion criteria for SCA3 patients were (1) known recessive X‐linked and mitochondrial ataxias, (2) exclusion of SCA3 diagnosis by previous genetic tests, (3) asymptomatic patients and homozygotes of SCA3, (4) accompanying ailments that affect the SARA scores or other diseases associated with balance impairments such as musculoskeletal impairments, and (5) patients who were not able to stand without holding hand rails. The control participants were individually matched to each patient based on age, gender, and environmental characteristics. Relatives at risk for SCA3 were excluded from the control group. The study was approved by the Ethics Committee of the First Affiliated Hospital of Fujian Medical University. Written informed consent was obtained from each participant.

### Genotype and phenotype analyses

Genotyping was performed to confirm the diagnosis of SCA3 and determine the CAG repeat lengths. Genomic DNA was extracted from peripheral blood using a QIAamp DNA Blood Minikit (QIAGEN, Hilden, Germany) following standard procedures. We performed polymerase chain reaction (PCR) amplification combined with Sanger sequencing to determine the length of CAG repeats as previously reported.^20^

Four phenotypic measures were obtained from each patient by Dr. Gan and Dr. Xu who were both ataxia specialists: severity of ataxia, age at onset (AAO), disease duration, and progression rate of ataxia. The severity of ataxia was determined using the SARA, which is often used clinically to evaluate the severity and progression of SCA3.^10^ SARA is a rating tool comprised of eight cerebellar function tests with a total score ranging from 0 to 40 where higher scores reflect greater functional impairment. Score ranges between 3 to 7, 8 to 14, and more than 14 were used to split patients into three stages of mild, moderate, and severe.^21^ AAO was defined as the age when symptoms related to SCA3 first appeared, which was estimated according to the reports of patients, close relatives, or care providers. The disease duration indicated the time span between AAO and the age at first visit. Progression rate of ataxia was calculated as the SARA score divided by duration.

### Posturography Assessment

After the clinical evaluation, the patients’ posturographic evaluation was assessed at the same day by Dr. Liu who was a rehabilitation specialist and was blind to the patients’ clinical phenotypes. Balance was tested for both control and patient groups with a posturographic platform (Prokin 254 (Pro‐Kin Software Stability), TecnoBody S. r. l., Dalmine, 24044 Bergamo, Italy), using standardized methods.^22^ The details of the platform are described in detail elsewhere.^15^ The platform was used to measure both static and dynamic stability. The descriptions and clinical implications for all platform measures are presented in Table 1.

**Table 1:** The descriptions and clinical implications of platform measures.

Posturographic measure	Definition	Clinical implication
The postural parameters in static stability		
COP	The point of application of forces exchanged between feet and ground	Reflecting the capacity for active body control
Sway range SD (mm)	The mean error of COP	Larger values indicate poorer postural stability and greater body sway
Velocity of body sway (mm/s)	The mean velocity of COP	
Total sway area (mm^2^)	The area ellipse containing 90% of the sampled positions of the COP	
Total sway perimeter (mm)	The layout of a line connecting the different positions of the COP	
The postural parameters in dynamic stability		
LOS (%)	An individual’s weight‐shifting ability and voluntary limits of stability to 8 directional targets	Reflecting the interlimb coordination based on different task requirements in different directions
OBI (°)	The total variance in displacement from the center of the platform	Reflecting the ability of neuromuscular control

COP, center of foot pressure; LOS, limits of stability; OBI, overall balance index; SD, standard deviation.

Static stability is measured using the center of foot pressure (COP), which is defined as the point of application of forces exchanged between feet and ground. To measure COP, real‐time position and movement tracks are monitored^23^ and two key parameters are measured: sway range standard deviation (SD), and velocity of body sway. These two parameters are measured in the anterior‐posterior (AP) and medial‐lateral (ML) axes. In order to quantify the dispersion of COP measurements, we also included two additional parameters, total sway area, and total sway perimeter.^17^ The sway range SD and velocity of body sway are defined as the mean error and velocity of COP, respectively. The total sway area is the area ellipse containing 90% of the sampled positions of the COP, and the total sway perimeter is measured using the layout of a line connecting the different positions of the COP. These measures all reflect the capacity for active body control, and for all variables larger values indicate poorer postural stability and greater body sway.^16^ Since postural control requires visual processing,^24^ these six measurements were assessed in two visual feedback conditions, eyes open (EO) and eyes closed (EC), where the platform is the main source of visual feedback. In total, COP was measured using 12 outcome variables based on four parameters.

Dynamic stability was evaluated using limits of stability (LOS) and overall balance index (OBI). The LOS test assesses an individual’s weight‐shifting ability and voluntary limits of stability to eight directional targets.^25^ A lower LOS score indicates increased risk of falling. OBI is measured as the total variance in displacement from the center of the platform, based on previously published calculations.^26^ OBI reflects the ability to control dynamic balance, and a larger OBI indicates bigger fluctuations and therefore poorer postural control.^27^

### Statistical analysis

The normality of the distribution of all variables (clinical variables and postural parameters) was assessed using Kolmogorov–Smirnov tests. Following the results of these tests, variables with normal distribution and variables in non‐normal distribution were expressed as mean ± SD and median (range), respectively. Two‐independent sample *t*‐tests and Mann–Whitney *U* tests were used to analyze normally and non‐normally distributed measures, respectively. Key comparisons were SCA3 patients versus healthy control groups, and EO versus EC conditions. Chi‐squared tests were used to assess the gender difference between SCA3 patients and healthy control groups. Spearman’s rho test was used to analyze the direct correlation of each parameter with SARA scores, static parameters with SARA subscores of stance, and dynamic parameters with SARA subscores of gait. *P* values and values of the coefficient (*r*) from Spearman’s rho test were used to determine the parameters which have the most or the least effect on distinguishing ataxia.

Because of the large number of postural variables and the likelihood that they are intercorrelated, we performed a Principal Component Analysis (PCA) with varimax rotation in an unsupervised fashion as a method of dimension reduction,^28^ the method which has been previously used to evaluate gait pattern.^29^ To test whether our variables were suitable for PCA, we applied the Kaiser–Meyer–Olkin (KMO) test (where the KMO measure of sampling adequacy> 0.5 was considered to be suitable) and Bartlett’s test of sphericity (*P* < 0.05 was considered to be suitable).^30^ To reconstruct a lower dimensional dataset which still includes adequate sources of variance, we applied the Guttmane–Kaiser Eigenvalue greater‐than‐one rule and confirmed the results using a scree plot.^31^ Factor loadings greater than 0.512 were accepted when determining which items (variables) could be considered to load on a given factor.^32^ The resulting principal components (PCs) were used as measures of balance function and analyzed using Hotelling’s *T*‐squared tests to explore differences in balance function between patients and controls.

We used multivariable linear regression to examine the relationship between postural measures and phenotype variability in SCA3 patients. First, we assessed the predictors of the two balance PCs that resulted from the PCA analysis, static stability (PC1), and dynamic stability (PC2). Stability measures were predicted by the following independent variables: Gender (binary), AAO, disease duration, SARA score, and the CAG repeats length in normal alleles and expanded alleles. Second, we assessed predictors of the severity of ataxia as measured with the SARA score, using the following independent variables: PC1, PC2, Gender (binary), AAO, and the length in normal and expanded CAG repeats. Finally, predictors of the progression of ataxia were assessed using the following independent variables: PC1, PC2, Gender (binary), AAO and the length in normal and expanded CAG repeats.

All statistical analyses were performed using SPSS version 20.0 (SPSS Inc., Chicago, IL, USA). All the graphs were produced using the Microcal Origin graphing program (Version 9.6, 2019, OriginLab Corporation, Northampton, Massachusetts). Statistical test outcomes were considered significant at *P* < 0.05. In the analytic comparison of postural variables between different groups, a Bonferroni correction was made to adjust for multiple comparisons. The significant *P* value of multiple comparison was based on the number of total comparisons in each analysis.

## Results

### Clinical evaluation

The demographic characteristics of the 62 SCA3 patients (male: 33, female: 29) and 62 healthy participants (male: 31, female: 31) are presented in Table 2. There were no significant differences in the distributions of age (*P* = 0.920) or gender (*P* = 0.719) between the patient and control groups. In SCA3 patients the mean AAO was 32.54 ± 9.19 years, the median progression rate of ataxia was 1.20 (0.20–4.30), the average disease duration was 7.86 ± 3.47 years, and the mean length of expanded alleles were 75.20 ± 2.83 and the median length of CAG repeats in normal alleles were 20.00 (13.00–44.00). The mean SARA scores were 8.91 ± 2.98, the median SARA subscores in stance and gait were 2.00 (0.00–5.00) and 2.00 (1.00–6.00), respectively. Based on the ranges of SARA score, there were 15 patients in mild stage, 44 patients in moderate stage, and three patients in severe stage.

**Table 2:** Demographic characteristics of study subjects

	SCA3 patients	Controls	*P* Value
Number	N = 62	N = 62	NA
Age, years	40.32 ± 9.22	42.00 (18–65)	0.920^1^
Gender, M/F	33/29	31/31	0.719^2^
Age at onset, years	32.54 ± 9.19	NA	
Progression	1.20 (0.20–4.30)	NA	
Disease duration, years	7.86 ± 3.47	NA	
Normal alleles	20.00 (13.00–44.00)	NA	
Expanded alleles	75.20 ± 2.83	NA	
SARA	8.91 ± 2.98	NA	
SARA_stance_	2.00 (0.00–5.00)	NA	
SARA_gait_	2.00 (1.00–6.00)	NA	
Severity stage^3^			
Mild	*N* = 15	NA	
Moderate	*N* = 44	NA	
Severe	*N* = 3	NA	

Variables with normal distribution were represented as the mean ± standard deviation; variables in non‐normal distribution were expressed as median (range).

N, number; NA, not applicable; SARA, Scale for the Assessment and Rating of Ataxia; SARA_gait_, gait subscore of SARA; SARA_stance_, stance subscore of SARA.

^1^ Mann–Whitney *U* test.

^2^ Chi‐squared tests.

^3^ Mild: 3 to 7 on the SARA score; Moderate: 8 to 14 on the SARA score; Severe: more than 14 on the SARA score.

### Posturography

**Figure 1:**
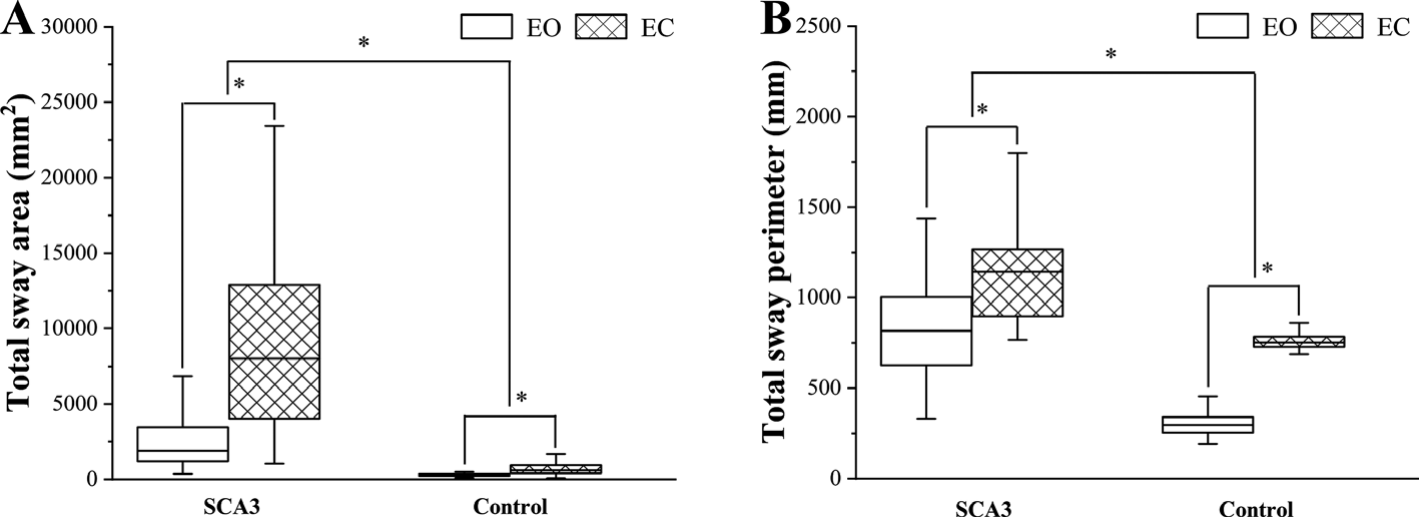
Total sway area and total sway perimeter plotted for EO and EC conditions for patients and controls. Statistical comparisons suggest that both total sway area (A) and total sway perimeter (B) are significantly higher in patients compared to controls, and in the EC compared to the EO condition, *P < 0.001.

There were a total of six group outcome variables derived from four parameters reflecting static stability. We first compared all outcome variables between patient and control groups, for the EO and EC conditions separately. We found that all outcome variables were significantly worse for patients compared to controls (all *ps* < 0.001). Second, we compared the values of all outcome variables between the EO and EC conditions, for patient and control groups separately. For all the variables, both patients and controls were significantly worse in the EC compared to the EO condition (all *p*s < 0.001). All outcome variables and statistical comparisons are summarized in Table 3, and the results clearly indicate that both SCA3 and a lack of visual feedback significantly impair static stability. The comparison of total sway area and total sway perimeter between patients and controls, and EO and EC is presented in Figure 1.

**Table 3:** Postural parameters in SCA3 patients and controls.

Posturographic measure	Visual conditions	Groups		*P* Value
		SCA3	Control	
The postural parameters in static stability				
Sway range SD in AP	EO	10.00 (4.33–32.67)	4.33 (2.33–9.00)	<0.001^1^
	EC	20.55 ± 7.98	6.67 (3.00–12.67)	<0.001^1^
	*P* Value	<0.001^1^	<0.001^1^	
Sway range SD in ML	EO	11.78 ± 4.64	4.00 (2.33–7.67)	<0.001^1^
	EC	22.08 ± 8.93	6.00 (2.67–15.00)	<0.001^1^
	*P* Value	<0.001^2^	<0.001^1^	
Velocity of body sway in AP	EO	21.83 (9.00–79.67)	8.00 (5.00–14.67)	<0.001^1^
	EC	60.33 (18.67–208.67)	13.00 (6.67–25.67)	<0.001^1^
	*P* Value	<0.001^1^	<0.001^1^	
Velocity of body sway in ML	EO	20.17 (7.67–52.33)	8.00 (5.00–17.33)	<0.001^1^
	EC	45.50 (11.33–158.33)	13.67 (6.67–26.00)	<0.001^1^
	*P* Value	<0.001^1^	<0.001^1^	
Total sway area	EO	1915.92 (360.00–14189.67)	299.67 (115.00–1154.33)	<0.001^1^
	EC	8244.00 (1121.33–47357.00)	690.00 (159.67–2980.33)	<0.001^1^
	*P* Value	<0.001^1^	<0.001^1^	
Total sway perimeter	EO	802.83 (331.33–2490.33)	296.33 (6.33–608.00)	<0.001^1^
	EC	2108.00 (565.00–6108.33)	510.33 (245.33–937.00)	<0.001^1^
	*P* Value	<0.001^1^	<0.001^1^	
The postural parameters in dynamic stability				
LOS		57.10 (21.27–87.70)	78.60 (40.20–156.33)	<0.001^1^
OBI		4.19 ± 0.91	2.85 (1.59–4.61)	<0.001^1^

Variables with normal distribution were represented as the mean ± standard deviation; variables in non‐normal distribution were expressed as median (range).

AP, anteroposterior; EC, eyes closed; EO, eyes open; LOS, limits of stability; ML, mediolateral; OBI, overall balance index; SD, standard deviation.

A Bonferroni correction was made to adjust for multiple comparisons, *P* < 0.002 was considered statistically significant (26 comparisons in total).

^1^ Mann–Whitney *U* test.

^2^ Two‐independent sample *t*‐tests.

**Figure 2:**
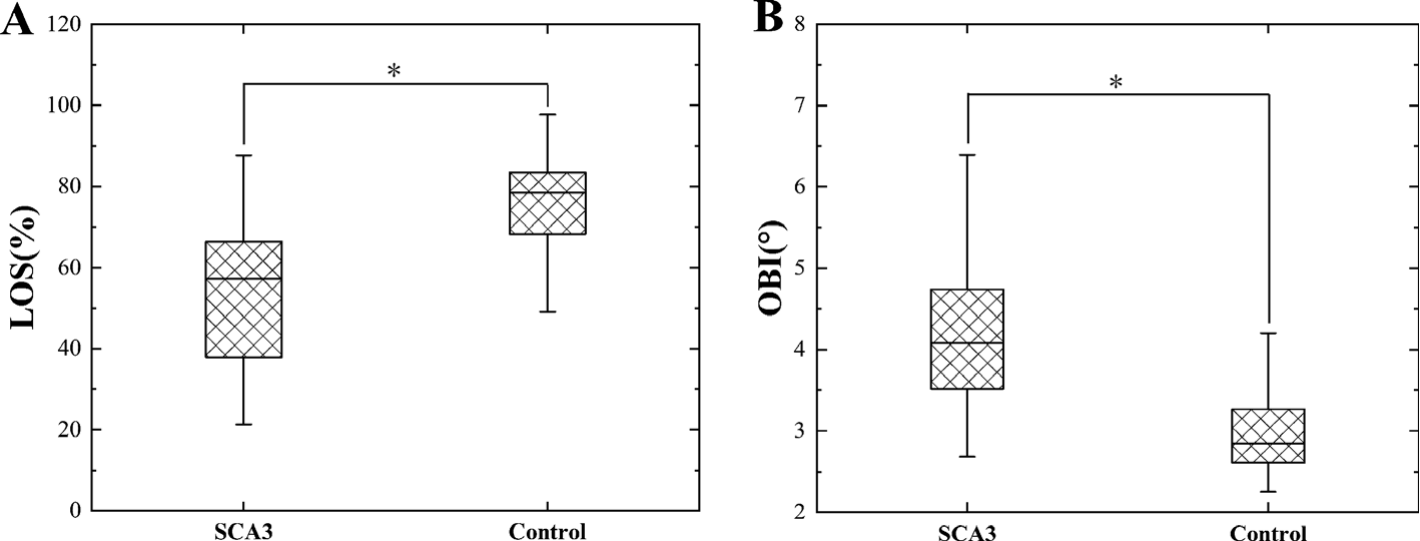
The comparison of LOS and OBI between patients and controls. (A) LOS values were significantly higher in controls compared to patients; (B) OBI values were significantly higher in patients compared to controls, *P < 0.001.

Following the analysis of static stability, we performed group comparisons for the dynamic stability measures, which are summarized in Table 3 and Figure 2. LOS values were significantly lower (suggesting impaired balance and increased risk of falling) in patients compared to controls (patients: 57.10 (21.27–87.70) vs. controls: 78.60 (40.20–156.33), *P* < 0.001), and the OBI values were significantly higher (suggesting poor postural control) in patients compared to controls (patients: 4.19 ± 0.91 vs. controls: 2.85 (1.59–4.61), *P* < 0.001).

In order to reveal these parameters change along the course of SCA3, we compared the values of all outcome variables between different stages. Since there were only three patients in the stage of severe, the values were compared only between stages of mild and moderate (Table 4). We found that all static parameters were significantly worse in moderate stage compared to mild stage (all *p*s < 0.001), whereas there was no significant difference in dynamic parameters between the two stages (LOS: *P* = 0.069; OBI: *P* = 0.271).

**Table 4:** Postural parameters along the course of SCA3.

	Stage^3^		*P*‐Value
	Mild	Moderate	
COP			
Sway range SD in AP with EO	6.91 ± 2.08	1067 (5.67–32.67)	<0.001^1^
Sway range SD in ML with EO	7.07 ± 1.85	13.04 ± 4.17	<0.001^2^
Velocity of body sway in AP with EO	16.75 ± 4.93	24.17 (11.67–79.67)	<0.001^1^
Velocity of body sway in ML with EO	15.58 ± 5.17	24.68 ± 9.75	<0.001^2^
Total sway area with EO	921.18 ± 438.16	2251.17 (554.00–14189.67)	<0.001^1^
Total sway perimeter with EO	606.44 ± 169.60	873.67 (360.33–2490.33)	<0.001^1^
Sway range SD in AP with EC	13.40 ± 3.92	22.66 ± 7.61	<0.001^2^
Sway range SD in ML with EC	13.42 ± 5.56	24.51 ± 7.91	<0.001^2^
Velocity of body sway in AP with EC	32.67 (18.67 ‐ 117.00)	69.67 (18.67–117.00)	<0.001^1^
Velocity of body sway in ML with EC	30.49 ± 10.19	49.00 (11.33–158.33)	<0.001^1^
Total sway area with EC	2710.67 (1121.33–14111.00)	9807.50 (1424.00–47357.00)	<0.001^1^
Total sway perimeter with EC	1088.00 (704.67–3205.67)	2285.50 (565.00–6108.33)	<0.001^1^
LOS	63.20 (30.23–75.97)	54.20 ± 16.29	0.069^1^
OBI	3.93 ± 0.97	4.22 ± 0.84	0.271^2^

Variables with normal distributions were represented as the mean ± standard deviation; variables in non‐normal distribution were expressed as median (range).

AP, anteroposterior; COP, center of foot pressure; EC, eyes closed; EO, eyes open; LOS, limits of stability; ML, mediolateral; OBI, overall balance index; SD, standard deviation.

A Bonferroni correction was made to adjust for multiple comparisons, *p* < 0.003 was considered statistically significant (14 tests in total).

^1^ Mann–Whitney *U* test.

^2^ Two‐independent sample *t*‐tests.

^3^ Mild: 3 to 7 on the SARA score; Moderate: 8 to 14 on the SARA score; Severe: more than 14 on the SARA score.

To analyze the direct correlation of each parameter with SARA scores, static parameters with SARA subscores of stance, and dynamic parameters with SARA subscores of gait, Spearman’s rho test was performed. We found that the most parameters significantly correlate with SARA scores, static parameters and dynamic parameters significantly correlate with stance subscores and gait subscores of SARA, respectively **(**Table 5 **)**. Based on the *p* values and values of the coefficient (*r*) from Spearman’s rho test, sway range SD in ML with EO (*r* = 0.737, *P* < 0.001) and total sway area with EO (*r* = 0.723, *P* < 0.001) were determined to be the most promising biomarker for ataxia; velocity of sway in AP with EC (*r* = 0.342, *P* = 0.010), total sway perimeter with EC (*r* = 0.374, *P* = 0.003), and OBI (*r* = 0.330, *P* = 0.009) were determined to have no effect in distinguishing ataxia.

**Table 5:** Correlations between SARA scores and postural parameters in SCA3

	SARA score^1^		SARA subscore^1^, ^2^	
	Coefficient	*P* Value	Coefficient	*P* Value
The postural parameters in static stability				
Sway range SD in AP with EO	0.658	<0.001	0.402	0.001
Sway range SD in ML with EO	0.737	<0.001	0.473	<0.001
Velocity of body sway in AP with EO	0.464	<0.001	0.281	0.027
Velocity of body sway in ML with EO	0.520	<0.001	0.358	0.004
Total sway area with EO	0.723	<0.001	0.460	<0.001
Total sway perimeter with EO	0.516	<0.001	0.325	0.010
Sway range SD in AP with EC	0.578	<0.001	0.466	<0.001
Sway range SD in ML with EC	0.626	<0.001	0.477	<0.001
Velocity of body sway in AP with EC	0.324	0.010	0.315	0.013
Velocity of body sway in ML with EC	0.439	<0.001	0.362	0.004
Total sway area with EC	0.623	<0.001	0.503	<0.001
Total sway perimeter with EC	0.374	0.003	0.308	0.015
The postural parameters in dynamic stability				
LOS	−0.459	<0.001	−0.420	0.001
OBI	0.330	0.009	0.325	0.010

AP, anteroposterior; COP, center of foot pressure; EC, eyes closed; EO, eyes open; LOS, limits of stability; ML, mediolateral; OBI, overall balance index; SARA, the Scale for the Assessment and Rating of AtaxiaSD, standard deviation.

A Bonferroni correction was made to adjust for multiple comparisons, *P* < 0.002 was considered statistically significant (28 tests in total).

^1^ Correlations were calculated with Spearman’s rho test.

^2^ The postural parameters in static stability were correlated to SARA subscores of stance, and the postural parameters in dynamic stability were correlated to SARA subscores of gait.

### Principal component analysis with postural parameters

**Figure 3:**
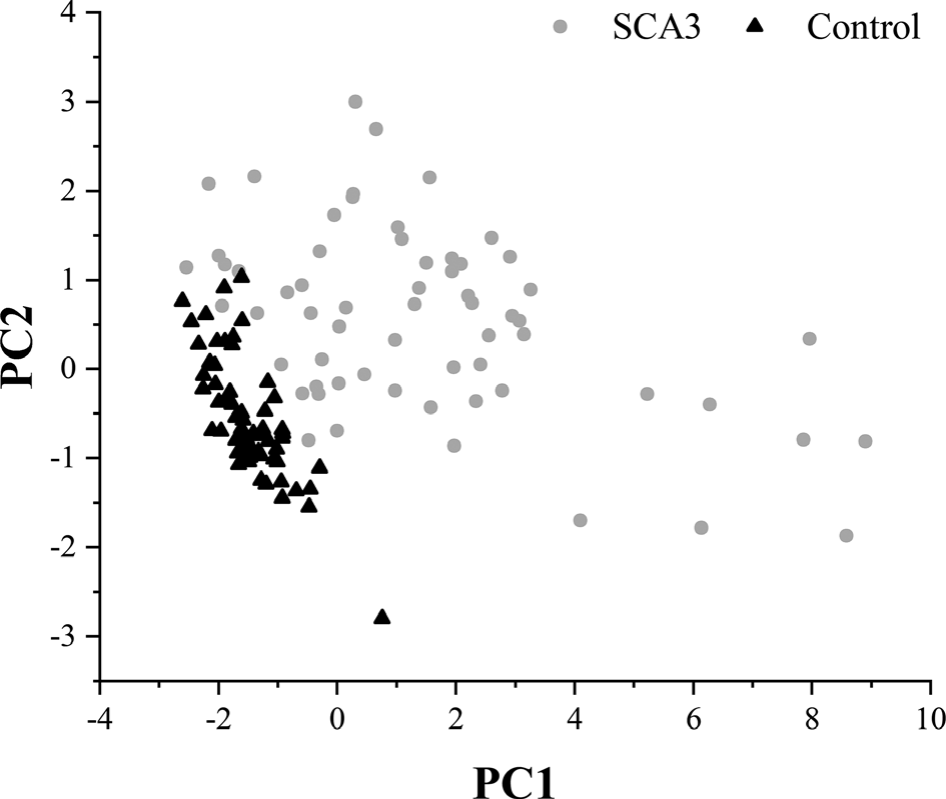
Scatter plot of PC1 and PC2 between SCA3 patients and controls. The scatter plot showed obvious separation between SCA3 patients and controls in PC1 (*P* < 0.001) and PC2 (*P* < 0.001), indicating that SCA3 patients had a distinct pattern of balance dysfunction compared with controls.

To determine the co‐occurrence of postural parameters, we performed PCA with varimax rotation to extract the relevant factors. We selected variables for inclusion in the PCA from amongst the 14 total static and dynamic stability measures, based in part on the results from the group and visual feedback comparisons. In particular, measures in the EC condition were more sensitive than those from the EO condition in differentiating patients from controls. Therefore, the PCA included six variables: four static stability variables from the EC condition (sway range SD in AP and ML directions, velocity of body sway in AP and ML directions, total sway area, and total sway perimeter), and the two dynamic stability measures, LOS and OBI. We determined the variables’ suitability for PCA using the KMO measure of sampling adequacy (0.768) and Bartlett’s test of sphericity (*P* < 0.001). We retrieved two components based on the Guttmane‐Kaiser Eigenvalue greater‐than‐one rule (confirmed using a scree plot), which accounted for 86.24% of the total variance. Based on the criteria of factor loading> 0.512, PC1 is the *static stability* factor, with high loadings for the six static stability variables, including the sway range SD in AP and ML directions, velocity of body sway in AP and ML directions, total sway area, and total sway perimeter. PC2 is the *dynamic stability* factor, with high loadings for the two dynamic stability variables, LOS, and OBI. Hotelling’s *T*‐squared tests revealed that factor scores from both PC1 and PC2 were significantly different between patients and controls (*ps* < 0.001, Fig. 3), in keeping with the results from the individual‐dependent measures.

### Relationship between postural instability and clinical manifestations

To examine the relationship between postural measures and clinical characteristics in patients, we performed a series of multivariable linear regressions. First, we investigated the predictors of postural instability (Table 6) in regression models predicting static and dynamic stability. Results indicated that increasing SARA scores predict poorer stability for both static stability (*β* = 0.297, *P* = 0.014) and dynamic stability (*β* = 0.172, *P* = 0.001), whereas higher CAG repeats length in expanded alleles did not predict poorer static stability (*β* = −0.350, *P* = 0.057) and dynamic stability (*β* = 0.012, *P* = 0.880). Gender also predicted postural stability, with being female as a possible risk factor for poorer static stability (*β* = 0.800, *P* = 0.044), and being male as a possible risk factor for poorer dynamic stability (*β* = 0.531, *P* = 0.003).

**Table 6:** The influences factors on balance function

	Coefficient estimate	Standard error	*P* Value
The influences factors on static stability^1^			
Gender^2^	−0.800	0.392	**0.044**
AAO	0.060	0.050	0.232
Disease duration	0.142	0.090	0.118
SARA	0.297	0.118	**0.014**
Normal CAG repeats	−0.026	0.038	0.495
Expanded CAG repeats	−0.350	0.174	0.057
The influences factors on dynamic stability^1^			
Gender^2^	0.531	0.174	**0.003**
AAO	−0.015	0.022	0.491
Disease duration	−0.060	0.040	0.134
SARA	0.172	0.052	**0.001**
Normal CAG repeats	0.021	0.017	0.209
Expanded CAG repeats	0.012	0.077	0.880

AAO, age at onset; SARS, Scale for the Assessment and Rating of Ataxia.

Bold value showed significance.

^1^ Balance function: static stability was measured by PC1 and dynamic stability was measured by PC2

^2^ Male versus female.

Next, we performed multivariable linear regression to investigate how static and dynamic stability factors predict measures of ataxia severity and progression of ataxia **(**Table 7). Results indicated that increased ataxia severity was predicted by poorer static stability (*β* = 0.690, *P* < 0.001), poorer dynamic stability (*β* = 1.727, *P* < 0.001), being female (*β* = −1.487, *P* = 0.018), and higher CAG repeats length in expanded alleles (*β* = 0.650, *P* < 0.001). In contrast, there were no significant predictors of ataxia progression.

**Table 7:** The influences of balance function on severity and progression of ataxia

	Coefficient estimate	Standard error	*P* Value
The influences of balance function on ataxia severity			
Gender^1^	−1.487	0.608	**0.018**
AAO	0.067	0.050	0.192
Normal CAG repeats	−0.008	0.041	0.844
Expanded CAG repeats	0.650	0.165	**<0.001**
Static stability^2^	0.690	0.136	**<0.001**
Dynamic stability^2^	1.727	0.352	**<0.001**
The influences of balance function on ataxia progression rate^3^			
Gender^1^	−0.095	0.246	0.701
AAO	0.023	0.020	0.273
Normal CAG repeats	−0.003	0.017	0.847
Expanded CAG repeats	0.053	0.067	0.428
Static stability^2^	−0.038	0.055	0.486
Dynamic stability^2^	0.087	0.142	0.544

AAO, age at onset.

Bold value showed significance.

^1^ Male versus female

^2^ Balance function: static stability was measured by PC1 and dynamic stability was measured by PC2

^3^ Ataxia progression rate: the SARA scores divided by disease duration (in years)

## Discussion

Results of this study demonstrate that SCA3 patients have clear impairments in postural control compared with age‐ and gender‐matched controls. Both static and dynamic stability were affected by clinical status (patient vs control) and static stability was also affected by degree of visual feedback. Among all the parameters, sway range SD in ML with EO and total sway area with EO were the most promising biomarker for ataxia, whereas velocity of sway in AP with EC, total sway perimeter with EC, and OBI had no effect in distinguishing ataxia. Moreover, static postural instability rather than dynamic postural instability deteriorated as the disease developing. Finally, within the patient group, regression analyses revealed close associations between postural measures and patient characteristics: static stability, dynamic stability, and expanded CAG repeats length all predicted the severity of SCA3.

Posturographic platforms have been useful in providing objective evaluation of postural stability deficits in neurodegenerative diseases such as Parkinson’s disease^15^, ^17^, ^23^, ^33^, ^34^, ^35^ and Multiple Sclerosis.^36^, ^37^ Outside of SCA3, this method has also been used to assess postural instability in other subtypes of spinocerebellar ataxia. Previous findings indicated that lower LOS scores were the best predictor of balance dysfunction in SCA1,^38^ and faster body sway was linked to impaired balance in SCA6.^39^ J. Schwabova *et al*. suggested the ML deviation is the only reliable marker for differentiating SCA2 and FRDA patients.^40^ In addition, many studies have used balance equipment to identify postural stability changes during preclinical stages of SCA.^41^, ^42^, ^43^ However, the current use of the Pro‐kin Balance system is its first application to understanding postural stability in SCA3 patients, and there are few other sources of quantitative posturography data in SCA3. Our observations support the future use of this quantitative approach, which provides objective measures that are sensitive to even small changes in neurological function of SCA3 patients. In future, these methods could be applied to develop indices for SCA3 postural instability which can quantify patients’ risk of falling.

The specific pathogenesis of postural instability has not been thoroughly revealed. The ability to maintain balance depends on the integration of the proprioceptive, vestibular, and visual systems, which are all important for movement control.^25^ Studies show that cerebellar damage results in deficits of proprioception, which is critical for retaining quiet standing.^19^, ^44^ Proprioceptive dysfunction can not only impair adaption to a changing base‐of‐support, but also reduces the perception of trunk, surface orientation, and postural sway in stance.^33^ This provides a possible mechanism for why SCA3 patients show higher body sway than controls. Therefore, we suggest that rehabilitation of proprioceptive‐motor training could be used to improve postural instability in SCA3 patients. The visual system provides information to the central nervous system regarding the position of the body relative to the environment. Visual input may compensate for the loss of somatosensory function and facilitate neural motor programs, even in healthy people.^42^ Impaired central processing of proprioceptive information contributes to postural instability, resulting in increased reliance on vision to maintain balance. In support of this mechanism, the current results suggest that visual input has significant influence on postural balance. Several investigators have used visual feedback to improve standing posture and keep postural control as steady as possible.^33^ Therefore, other exteroceptive stimuli, along with visual cues, may be required for balance training in SCA3 patients.

Future research would benefit from an expansion of the current method and the implementation of a longitudinal study. First, while the parameters we chose provided reliable assessments of static and dynamic stability,^17^ we only used six parameters including eight variables for this study from a much wider range of potential measures available in the Pro‐kin system. Second, our group comparison is cross‐sectional and the evaluation of disease progression is retrospective; the predictive validity of key measures remains to be explored in terms of monitoring the progression of disease in a longitudinal study; Third, the common nonataxia symptoms of SCA3, such as peripheral neuropathy, dystonia, and parkinsonism, may affect postural stability. However, these nonataxia symptoms were unavailable at this study. Further study will be needed to explore the influence of nonataxia on the postural parameters.

In conclusion, this study supports the use of the Pro‐kin system to detect postural instability in SCA3 patients. Stance posturography is a promising tool in the development of balance rehabilitation strategies, which can be used to measure functional improvements in response to therapeutic interventions.

## Conflict of Interest

All authors report no conflicts of interest relevant to this work.

## Authors’ Contributions

Dr. X‐H Liu carried out study concept and design, acquisition of data, statistical analysis and interpretation, writing of the manuscript, and critical revision of the manuscript for important intellectual content. Dr. Y Li involved in study concept and design, statistical analysis and interpretation, and writing of the manuscript. Dr. H‐L Xu involved in study concept and design, acquisition of data, statistical analysis, and interpretation. Dr. A Sikandar, Dr. W‐H Lin, Dr. G‐H Li, Li Dr. S‐B Yu, Dr. X‐F Li, Dr. A Alimu, and Dr. X‐H Ye involved in acquisition of data. Dr. N Wang, Dr. J Ni, and Dr. W‐J Chen involved in study concept and design and acquisition of data. Dr. S‐R Gan contributed to study concept and design, acquisition of data, analysis and interpretation, and critical revision of the manuscript for important intellectual content.
